# Dynamic regulation of macrophage polarization via coupled multicellular networks

**DOI:** 10.1186/2051-1426-1-S1-P182

**Published:** 2013-11-07

**Authors:** Yishan Chuang, Joshua N Leonard

**Affiliations:** 1 Chemical and Biological Engineering, Northwestern University, Evanston, IL, USA

## 

Macrophages functionally polarize towards either immunostimulatory (M1) or immunosuppressive (M2) phenotypes, and tumor-associated macrophages (TAMs) exhibit an M2-like phenotype that impedes immunotherapy. Although the importance of M2 cells in tumor progression is well established, the process by which macrophages “decide” to adopt an M2 phenotype in complex environments is not well understood and is the subject of this study. Most prior investigations have applied “coherent” stimuli (exclusively pro-M1 or pro-M2 signals), yet these stimuli rarely exist independently in vivo. To understand how macrophages integrate incoherent stimuli (both pro-M1 and pro-M2 simultaneously), we first exposed macrophages to dosed combinations of IL-10 and IL-12. Contrary to reports that IL-12 directly alters macrophage polarization state, we observed that when competing directly with IL-10, IL-12 had little direct effect on macrophage polarization. Instead, our data suggest that maintenance of the IL-10-induced M2 state is mediated via the NFκB inhibitor BCL-3, and the M2 state was disrupted by either removal of IL-10 or co-treatment with the pro-M1 stimulus IFNγ. Together, these data suggest that IL-12 may indirectly promote M1 phenotypes by inducing innate and adaptive immune cells to produce IFNγ in the tumor microenvironment. To investigate how interactions between macrophages may influence polarization outcomes, we next examined polarization in individual cells using flow cytometry. Surprisingly, we observed that M1 and M2 cells co-existed in vitro, and that the probability of polarization towards M2 was dose-dependent on IL-10 and independent of IL-12 co-treatment. These data represent the first evidence to date the macrophage polarization is “stochastic” at a coarse-grained level. Heterogeneity was pronounced immediately following activation via LPS and evolved via dynamics that differed based upon cytokine pretreatment. Finally, we investigated macrophage polarization dynamics in vivo. Although conventional wisdom states that macrophages transition from M1 to M2 during tumor progression, this putative transition is poorly understood and sparsely characterized at early stages of tumor development. Therefore, we characterized the dynamics of macrophage polarization during melanoma progression in a syngeneic murine model (B16F0 cells and C57/Bl6 mice). Heterogeneous macrophage polarization was observed at early time points post-tumor implantation, macrophages transitioned from M1 to M2 (systemically and at the tumor) as CD8+ CTL transitioned into regulatory T cell phenotypes. Such insights reshape our understanding of immune responses and should help to identify novel therapeutic targets and strategies for treating cancer.

